# Characterization of mental health in cannabis dispensary users, using structured clinical interviews and standardized assessment instruments

**DOI:** 10.1186/s12888-019-2324-z

**Published:** 2019-11-01

**Authors:** Jade C. Yau, Shu Min Yu, William J. Panenka, Hadley Pearce, Kristina M. Gicas, Ric M. Procyshyn, Caroline MacCallum, William G. Honer, Alasdair M. Barr

**Affiliations:** 1 Department of Anesthesiology, Pharmacology & Therapeutics, University of British Columbia, 2176 Health Sciences Mall, Vancouver, British Columbia V6T 1Z3 Canada; 2 Department of Psychiatry, University of British Columbia, Vancouver, British Columbia Canada; 3 Department of Psychology, York University, Toronto, Ontario Canada; 4 Department of Medicine, University of British Columbia, Vancouver, British Columbia Canada

**Keywords:** Cannabis, Clinical interview, Dispensary, Mental health, MINI, Psychiatric symptoms

## Abstract

**Background:**

Cannabis is commonly used for its medical properties. In particular, cannabis is purported to have beneficial effects on a wide range of neuropsychiatric conditions. Studies assessing mental health in cannabis dispensary users typically evaluate symptoms using self-report check lists, which provide limited information about symptom severity, and whether subjects meet criteria for a psychiatric diagnosis. There is, therefore, a need for studies which assess mental health in dispensary users with standardized and well validated scientific instruments, such as those used in clinical drug trials.

**Methods:**

One hundred medical cannabis users were recruited from a community dispensary. All subjects completed a structured clinical interview with the Mini-International Neuropsychiatric Interview (MINI). Subjects also completed the Perceived Stress Scale-10, PROMIS Fatigue Scale, PROMIS Sleep Disturbance Scale, Beck Depression Inventory, the Patient Health Questionnaire-15 and the Brief Pain Inventory. Details about cannabis use were also recorded.

**Results:**

Lifetime prevalence of mental illness in this cohort was high, and a large proportion of subjects endorsed psychological symptoms. The proportion of subjects who met criteria for classification of a current psychiatric disorder was low for mood disorders, but high for anxiety disorders and substance abuse/dependence. Cannabis use differed between the main psychiatric conditions.

**Conclusions:**

The present results indicate that rates of mental illness may be high in medical cannabis dispensary users. Use of structured clinical assessments combined with standardized symptom severity questionnaires provide a feasible way to provide a more rigorous and detailed evaluation of conditions and symptoms in this population.

## Background

Cannabis is consistently reported as one of the most frequently used drugs worldwide [1]. While it is used predominantly for recreational purposes, there is an increasing body of evidence which indicates that cannabis and its constituent cannabinoids hold therapeutic potential for a wide variety of medical conditions [2]. In particular, cannabis is commonly used to medicate neuropsychiatric symptoms, including pain, insomnia, anxiety, depression and many other disorders that are related to mental health [3–6]. However, the relationship between cannabis use and mental health is complex, and there are multiple reports that cannabis use is also associated with greater anxiety [7], depression [8] and psychosis [9, 10]. With this in mind, the potential role of cannabis for medical purposes (and especially for neuropsychiatric conditions) should be further clarified, as cannabis gains interest as a supplemental or alternative treatment to conventional medications, and increasingly becomes decriminalised in many parts of the world [11, 12].

One especially common source of medical cannabis for users in Western countries is through community dispensaries [13–15], where clients are able to purchase a range of cannabis products. Recent studies have therefore conducted self-report surveys with this population to better understand the specific conditions and symptoms that are being targeted by medical cannabis users. These studies have been invaluable in furthering our understanding of medical cannabis use in the general population, and have reinforced the point that the majority of medical cannabis users consume the product for neuropsychiatric conditions [16–18]. However, self-report check-list surveys typically provide limited granularity of mental health symptoms, and symptom severity is rarely quantified.

Thus, there is a need for studies which assess mental health in dispensary users with standardized and well validated scientific instruments, such as those used in clinical drug trials. This will significantly extend the literature by providing an in-depth characterization of the neuropsychiatric features of medical cannabis users with implications for improved medical management. The goal of the present study was therefore to measure mental health and psychiatric symptom severity in a non-epidemiological sample of medical cannabis users from a dispensary in Vancouver, Canada. A structured clinical interview was completed to determine whether participants met criteria for a current or lifetime diagnosis of a major psychiatric disorder. In addition, subjects completed seven standardized questionnaires to quantify symptom severity in the domains of stress, fatigue, insomnia, pain, depression, somatic symptoms and nicotine dependence. These seven diverse questionnaires were chosen because medical cannabis is used for a broad spectrum of conditions related to mental health, and this will often include symptoms that are not included in the diagnoses of specific psychiatric disorders (such as stress) but which contribute significantly to overall mental health and quality of life, and represent a major reason for using medical cannabis. Our hypothesis was that we would identify subjects both with valid psychiatric disorders as well as many individuals who did not meet criteria for a psychiatric disorder, yet had a wide range of mental health issues, and therefore the questionnaires were chosen to capture and quantify the diverse range of these sub-syndromal symptoms. Findings were combined with extensive data on cannabis use to provide a detailed picture of mental health in the dispensary population.

## Methods

### Study population

Participants (*n* = 100) were consecutively recruited exclusively from the Evergreen Cannabis Society compassion club, which is a cannabis dispensary in Vancouver, Canada. Participants were eligible for the study if they were 19 years old or older, a current member of Evergreen, and able to give informed consent. All subjects provided written informed consent. The study was approved by the Behavioural Research Ethics Board of the University of British Columbia (protocol H16–01830). Subjects were recruited through flyers at Evergreen advertising a study on the medical benefits of cannabis. Participants were given an honorarium of $50 for their time, which took approximately 4 h to complete per subject (including time to complete subject consenting, fill out the questionnaires and conduct the Mini-International Neuropsychiatric Interview (MINI). The honorarium was provided by the graduate student (JCY) at the end of the interview. Once subjects had been consented, all subjects completed the interviews and none withdrew. No subjects were excluded during the study, for reasons such as obvious intoxication or being a non-cannabis user.

### Measures

Detailed demographic information was collected, including age, gender, marital status, living status, education, and current employment. Specific questions regarding cannabis characteristics were also recorded, including age of first cannabis use, conditions or symptoms for which cannabis was used medically, preferred consumption method, frequency and time of day of cannabis use, amount of cannabis consumed, preferred cannabinoid content and any negative experiences from using cannabis. Data for these and other forms was uploaded onto a password-controlled, encrypted laptop and then transferred to the secure servers at British Columbia Children’s Hospital.

Six self-report questionnaires were administered, in order to obtain detailed information about the individual’s general well-being and mental health. These included the Perceived Stress Scale 10 (PSS10), Patient Reported Outcomes Measurement Information System (PROMIS) Fatigue Scale, PROMIS Sleep Disturbance Scale, Beck Depression Inventory-II (BDI-II), the Patient Health Questionnaire 15 (PHQ-15) and the Brief Pain Inventory (BPI). Participants who indicated that they currently smoke cigarettes also completed the Fagerström Test for Nicotine Dependence (FTND). All seven questionnaires are used commonly in the field of clinical research, and each has been well validated and determined to be reliable [19–25]. The questionnaires were all administered and collected by the graduate student (JCY) and completed in a quiet, private space at the dispensary.

The PSS10 enables the participant to rate their life in terms of unpredictability, lack of control and stress overload [19]. It consists of 10 items, which include 6 positively phrased items and 4 negatively phrased items that are rated on a 5-point Likert scale. Total score indicates levels of perceived stress. The PROMIS fatigue scale rates how tired participants were over the past 7 days [26]. Items on the fatigue scale are split into “experience of fatigue” - specifically the frequency, duration and intensity - as well as the physical, mental and social effects the fatigue caused. The PROMIS sleep disturbance scale is an 8-item measure for sleep disturbance in adults, during the past 7 days [26]. Both the PROMIS Fatigue and Sleep Disturbance scales are rated on a 5-point scale. The BDI-II is a self-report rating inventory with 21-items that evaluates depressive symptoms and attitudes over the past 2 weeks [27]. Total scores categorize severity of depression: 1–13 = normal; 14–19 = mild depression; 20–28 = moderate depression; 29–63 = severe depression. The PHQ-15 is a diagnostic tool that measures 15 somatic symptoms [28]. Total PHQ-15 scores of 5, 10, and 15 signify cut-off points for low, medium and high severity of somatic symptoms, respectively. The BPI evaluates pain severity and the impact that pain has on daily functioning [29]. In the first question, participants select if they had any pain in specific areas of their body beyond regular aches and pain, and only complete the remainder of the pain questionnaire if this is the case. They then rate their worst, least, average and current pain intensity. They also rate the degree of interference the pain has with general activity. The FTND assesses addiction to nicotine [30]. Dependency levels are categorized as low (0–2 points), average (3–5 points), strong (6–7 points) to very strong (8–10 points).

### MINI

In order to determine whether participants met criteria for a psychiatric diagnosis, the MINI version 6 was administered. It is a structured clinical interview used to make diagnoses of psychiatric disorders based on the Diagnostic and Statistical Manual of Mental Disorders, 4th edition (DSM-IV) and the International Classification of Diseases, 10th edition (ICD-10) [31]. All MINIs were conducted by the graduate student (JCY), who was trained extensively in how to conduct the interview, as per our ongoing studies with cohorts with mental health and addiction issues [32–37]. The MINI was conducted in a quiet, private space at the cannabis dispensary.

ICD-10 codes for diagnoses captured in this version of the MINI are: Depressive episode: F32; Mild depressive episode: F32.0; Moderate depressive episode: F32.1; Severe depressive episode without psychotic symptoms: F32.2; Severe depressive episode with psychotic symptoms: F32.3; Other depressive episodes: F32.8; Depressive episode, unspecified: F32.9; Recurrent depressive disorder: F33; Manic episode: F30; Hypomania: F30.0; Mania without psychotic symptoms: F30.1; Mania with psychotic symptoms: F30.2; Other manic episodes: F30.8; Manic episode, unspecified F30.9; Bipolar affective disorder: F31; Bipolar affective disorder, current episode hypomanic: F31.0; Bipolar affective disorder, current episode manic without psychotic symptoms: F31.1; Bipolar affective disorder, current episode manic with psychotic symptoms: F31.2; Bipolar affective disorder, current episode mild or moderate depression: F31.3; Bipolar affective disorder, current episode severe depression without psychotic symptoms: F31.5; Bipolar affective disorder, current episode mixed F31.6; Other bipolar affective disorders: F31.8; Bipolar affective disorder, unspecified: F31.9; Suicidality: Suicide attempt: T14.91; Suicidal ideation: R45.851; Personal history of self-harm (suicide attempt): Z91.5; Intentional self-harm suicide attempt: X60-X84; Anxiety: Agoraphobia: F40.0; Social phobia: F40.1; Panic disorder: F41.0; Generalized anxiety disorder: F41.1; Post-traumatic stress disorder: F43.1; Other specified anxiety disorders: F41.8; Obsessive-compulsive disorder: F42; Drug Abuse: Alcohol dependence: F10.20; Alcohol abuse: F10.10; Other psychoactive substance abuse (uncomplicated): F19.10; Other psychoactive substance dependence (uncomplicated): F19.20; Miscellaneous: Acute and transient psychotic disorders: F23; Anorexia nervosa: F50.0; Bulimia nervosa: F50.2; Dissocial (antisocial) personality disorder: F60.2.

### Data analysis

Descriptive analysis was performed on participant demographics, cannabis characteristics, questionnaire results and MINI diagnoses. Continuous data were tested for normality with the Shapiro–Wilk test. The independent t-test was used for normally distributed continuous variables, whilst the Mann–Whitney U test was used for non-normal continuous variables. Categorical data were analysed using Chi-squared tests. All analyses were performed using the Statistical Package for the Social Sciences (SPSS) software version 24 (SPSS Inc., Armonk, USA).

## Results

### Descriptive

Descriptive analyses (Table 1) of the demographics indicated that participants were mostly male (68%), aged between 19 and 30 years (59%), single/never married (69%), and of Caucasian ethnicity (66%). Most lived with roommates/friends (45%) or spouse/significant other/children (26%), while the highest education level most participants achieved was a college degree (46%). Job classification varied widely, while participants worked most commonly full time/35h hours per week (33%).

**Table 1:** Demographic characteristics of subjects (*n* = 100) enrolled in the study

Characteristic	N
Gender	
Male	68
Female	32
Age	
18–24	35
25–30	24
31–35	10
36–40	9
41–45	2
46–50	7
51–55	8
56 and over	5
Ethnicity	
African, Caribbean	1
Caucasian	66
Asian	9
Hispanic	4
Middle Eastern	1
Mixed, Other	18
First Nations	1
Highest Education Level	
Less than high school	4
High School graduate or GED	13
Some college	32
College degree	46
Doctoral degree	1
Employment Status	
Part-time (34 h/week or less)	23
Full-time (35 h/week or more)	33
Student	15
Unemployed	9
Retired	5
Other	10

### MINI and psychiatric diagnoses

**Fig. 1:**
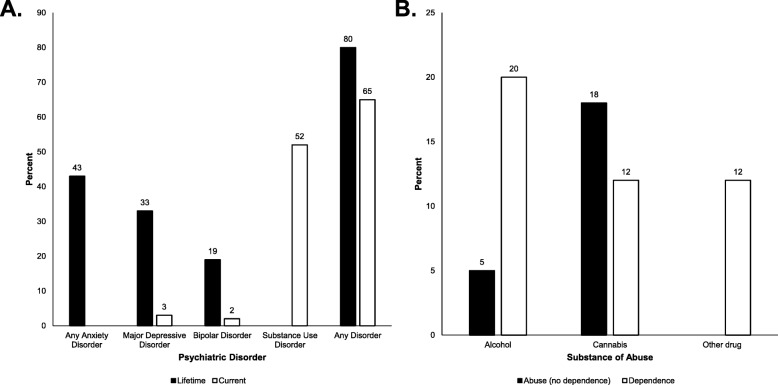
**a** Relative rates of major psychiatric disorders, based on diagnosis with the Mini International Neuropsychiatric Interview. Note that the MINI only evaluates for alcohol and substance abuse within the past 12 months, denoted here as “current” substance use disorder. “Any Anxiety Disorder” includes lifetime panic disorder, current agoraphobia, current social anxiety disorder, current obsessive compulsive disorder, current post-traumatic disorder, and current generalized anxiety disorder. **b** Types of substances causing substance use disorder. The bars depict the rates of dependence, and abuse that does not meet criteria for dependence; bars are clustered by the type of substance. The MINI evaluates for abuse of alcohol and non-alcoholic substances separately, therefore it is possible for a subject to be diagnosed with both an alcohol and non-alcoholic substance use disorder. “Other drugs” includes cocaine, heroin, methamphetamine, clonazepam, 3,4-methylenedioxymethamphetamine, and 3-fluorophenmetrazine

Based on the results of the MINI (Fig. 1), 50% of participants had experienced a Major Depressive Episode (MDE) in the past, and only 3% participants had a MDE currently. Similarly, 33% of participants were diagnosed with past Major Depressive Disorder (MDD) and only 3% with current MDD. Anxiety related disorders were relatively common, with a total of 43% of participants being diagnosed with any anxiety disorder; all anxiety disorders are current (past 6 months), as the MINI does not record past episodes. Non-alcohol substance dependence and substance abuse was diagnosed for 30 and 42% of participants, respectively, while alcohol dependence (20%) and alcohol abuse (25%) diagnoses were also noted. Diagnoses for drug dependence are current (within the past year) only for the MINI. In total, 80% subjects met criteria in the MINI for any lifetime diagnosis of at least one psychiatric disorder, and 65% met criteria for two or more disorders. Sixty six percent of subjects met criteria for at least one current psychiatric disorder, and 52% met criteria for at least two or more current disorders.

### Mental and general health

From the PSS10, 37% of subjects had low, 57% had moderate, and 5% had high perceived stress (Table 2). From the PHQ-15, 43% of participants ranked minimal, 32% ranked low, 21% ranked medium and 4% ranked high somatic symptoms. For the BPI, 35 participants answered that they felt additional pain, and mean scores of pain rating and pain interference were 14.7 (± 10.3) and 15.4 (± 12.4) respectively. Ninety eight percent of subjects completed the PROMIS sleep disturbance, with an average T-score of 46.7 (± 8.5). All participants completed the PROMIS fatigue questionnaire, with an average T-score of 51.1 (± 7.7). The BDI scores were mostly normal (83%), with 9% scoring mild depression, 7% as moderate depression and 1% are severe depression. Only regular smokers were eligible to complete the FTND (*n* = 21), which indicated that 47.6% ranked low nicotine dependence, 38.0% ranked average dependence, and 14.3% ranked high dependence.

**Table 2:** Scores for the 8 different symptom severity questionnaires administered to subjects in the study (*n* = 100). Analysis includes standard cut-off values recommended for categorization of symptoms into rankings of increasing severity

Scale Rank (Score)	N (out of 100 subjects)
Perceived Stress Scale^a^	
Low perceived stress (0–4)	37
Moderate perceived stress (14–26)	57
High perceived stress (27–40)	5
PROMIS Fatigue	
20 to 30	0
31 to 40	9
41 to 50	35
51 to 60	46
61 to 70	9
71 to 80	1
81 to 90	0
PROMIS Sleep Disturbance^b^	
20 to 30	4
31 to 40	18
41 to 50	42
51 to 60	29
61 to 70	4
71 to 80	1
Beck Depression Inventory	
Normal (0–10)	73
Mild mood disorder (11–16)	14
Borderline clinical depression (17–20)	6
Moderate depression (21–30)	7
Severe depression (31–40)	0
Extreme depression (over 40)	0
Patient Health Questionnaire^c^	
Minimal (0–4)	42
Low (5–9)	32
Medium (10–14)	21
High (15–30)	4
BPI Pain Rating^d^	
0 to 10	12
11 to 20	15
21 to 30	5
31 to 40	2
41 to 50	1
BPI Pain Interference^d^	
0 to 10	15
11 to 20	7
21 to 30	7
31 to 40	6
41 to 70	0
Fagerstrom Test for Nicotine Dependence^e^	
Low dependence (0–2)	10
Average dependence (3–5)	8
Strong dependence (6–7)	3
Very strong dependence (8–10)	0

^a^One subject did not complete the Perceived Stress Scale questionnaire

^b^Two subjects did not complete the PROMIS sleep Disturbance questionnaire

^c^One subject did not complete the Patient Health Questionnaire

^d^Only subjects who endorse question 1 of the BPI (*n* = 35) complete the remaining questions of the BPI

^e^Only regular smokers (n = 21) were eligible to complete the Fagerström test

### Cannabis use characteristics

Age of first cannabis use was most commonly 16–20 years (56%) (Table 3). Most subjects starting using cannabis regularly at ages 16–20. Reasons for starting medical cannabis included: “conventional treatments don’t work” (20%) and “wanted a natural treatment” (51%), which may anecdotally reflect the high rates of medication errors and side-effects associated with many psychiatric medications [38–40].

**Table 3:** Characteristics of cannabis use by subjects (*n* = 100) enrolled in the study

Cannabis Characteristic	
Age of First Cannabis Use (± SD)	17.3 (± 4.8)
Age of Onset for Regular Use (± SD)	20.4 (± 6.2)
Years of Medicinal Cannabis Use (± SD)	5.5 (± 7.1)
Initial intention of cannabis use	
Medicinal	11
Recreational	88
Ever smoked cigarettes	70
Current smoker	21
Reason for medical cannabis use	
Conventional treatments unhelpful	22
Prefer natural treatment	52
Doctor recommendation	10
Internet search for treatments	16
Hear-say from peers	40
Other	18
Mental health conditions to treat with cannabis	
Anxiety, stress	77
Depression	47
Insomnia, sleep issues	53
PTSD	14
Bipolar disorder	5
Focus, attention	14
Other	31
Using cannabis to treat other health conditions	25
Reasons conventional treatments not preferred	
Not effective	44
Side effects	54
Expensive	25
Other	25
Dispensary visits per month (± SD)	4.0 (± 3.4)
Frequency of cannabis use, times per month (± SD)	47.1 (± 30.8)
Amount of product use per week	
Dried cannabis	
<1 g	31
3.5 g (an eighth)	40
7 g (a quarter)	17
14 g (a half)	6
28 g (an ounce)	2
Capsules (1–2 daily)	10
Tincture (~ 5 drops daily)	4
Other (~ 5 teabags, 20 oz. creams, oils and bath salts)	6
Time of day usually use cannabis	
Early morning (6 am–9 am)	9
Mid-morning (9 am–11 am)	13
Midday (10 am-2 pm)	17
Mid-afternoon (2 pm–4 pm)	16
Early evening (4 pm–7 pm)	27
Evening (7 pm–10 pm)	67
Late night (10 pm-12 am)	35
Middle of the night (12 am–6 am)	10
On an as-needed basis	37
Preferred form of consumption	
Smoking	71
Vaporiser	43
Edibles	20
Capsules	17
Tinctures	15
*Tincture THC*	3
*Tincture CBD*	6
*Tincture Mix*	7
Tea	9
Topical	11
Cannabis products usually bought	
Dried cannabis	91
CBD capsules	23
Edibles	17
Extracts, concentrates	9
Tea	8
Tinctures	8
Massage oil, cream	13
Other	2
Preferred THC to CBD concentrations	
Pure THC	20
Pure CBD	18
High THC/Low CBD	35
Equal THC/CBD	35
Low THC/High CBD	21
No preference	6
Don’t know	14
Negative effects from medical cannabis	
Short-term memory loss	25
Dizziness	16
Decreased attention span	21
Decreased sensory perception	6
Increased anxiety	36
Paranoia	26
Impaired body movements	9
Difficulty breathing	8
Other	8
Frequency of negative effects	
Every time I use cannabis	2
Nearly every time	4
Sometimes	6
Rarely	20
Only during a certain cannabis type	19
Not since the first time I used cannabis	1
Other	10

Conditions treated with medical cannabis included most commonly (subjects could indicate as many as appropriate): anxiety/stress (77%), depression (47%) and insomnia (53%). When subjects were asked to choose one specific condition as their primary reason for using medical cannabis, anxiety (43%) was the most common condition. Other common primary conditions included insomnia/sleep issues (18%) and depression (16%). Common reasons against using conventional treatments included that they were “not effective” (40%), had side effects (50%), were expensive (23%), or other reasons (35%), such as “strong preference against using man-made pills” or “not a long-term solution”. Members of the Evergreen compassion club most often visited once a week (39%).

Smoking was the most preferred form of cannabis consumption (71%) and dried cannabis (91%) was the most commonly bought cannabis product. Subjects used cannabis most commonly in the evening. Preference for cannabinoid concentration was broad, with subjects expressing a preference for high tetrahydrocannabinol (THC) /low cannabidiol (CBD) (35%), equal THC/CBD (35%), low THC/high CBD (21%), pure THC (20%) and pure CBD (18%). Details about cannabinoids are provided in Table 4. Fifty five percent of participants experienced negative effects from medical cannabis, including anxiety (*n* = 36), paranoia (*n* = 26) and short-term memory loss (*n* = 25).

**Table 4:** Cannabinoid concentrations based on the top strains sold at the Evergreen Dispensary

Concentration	THC (w/w%)	CBD (w/w%)
Pure THC	24.3	–
High THC: Low CBD	24.5	1.8
Equal	9.8	8.6
Low THC: High CBD	0.7	16.1
Pure CBD	–	^a^

Concentrations are based on strains of the dried cannabis product. Strains include sativa, indica and hybrid strains. “Pure THC” strains were those with a high THC concentration and “nominal” CBD

^a^No CBD concentration could be reported for the “Pure CBD” concentration since Evergreen did not sell any dried cannabis with only CBD. It is very difficult to come up with dried plant material that is CBD-pure

### Specific psychiatric disorders

As a follow up, we conducted exploratory analyses of the characteristics of cannabis users with the three most common lifetime psychiatric disorders, as determined by the MINI, to see if there were differences in cannabis use between conditions (Table 5). These included lifetime depression, anxiety and substance abuse (excluding cannabis dependence) and were co-occurring in a number of cases. On the self-report questionnaires, both the anxiety and depression groups exhibited significantly greater scores on the PSS10, PROMIS Fatigue, BDI and PHQ-15 tests compared to those without these conditions in the cohort, while the substance abuse group had higher scores on the PSS10 and BDI. On cannabis use, the anxiety group was more likely than non-anxiety subjects to use tinctures or capsules, have a doctor recommendation for cannabis, use CBD capsules or pure CBD, use on an “as-needed” basis, and experience negative side-effects. The lifetime depression group had a higher proportion of females than the non-depressed group, were more likely to use CBD capsules, and use cannabis depending on symptoms. In addition, participants diagnosed with lifetime depression visited the dispensary more frequently, around two or three times a week, than participants without depression. The substance use disorder group were younger than non-substance users, used cannabis because they believed that conventional treatments do not work, visited the dispensary more often, and were more likely to smoke cannabis (but less likely to vape or use tinctures).

**Table 5:** Exploratory analyses of subjects who met criteria for the three most common psychiatric disorders based on the MINI interview. Analyses compared those who met criteria for the disorder to those who did not. For substance dependence, people who had only the cannabis form of dependence were excluded from analysis, as they were predicted a priori to have higher rates of cannabis use

	Anxiety Disorders			Major Depressive Disorder			Substance Use Disorder (Except Cannabis)		
	No (*N* = 57)	Yes (*N* = 43)	*P*-value	No (*N* = 67)	Yes (*N* = 33)	*P*-value	No (*N* = 48)	Yes (*N* = 22)	*P*-value
Male (%)	40 (70.2)	28 (65.1)	0.591	52 (77.6)	16 (48.5)	0.003	31 (64.6)	14 (63.6)	0.939
Current Age (± SD)	33.9 (± 12.3)	33.2 (± 13.9)	*0.502*	34.2 (± 13.6)	32.4 (± 12.1)	*0.536*	37.6 (± 13.7)	28.1 (± 10.3)	*0.003*
Current Smoker (%)	11 (19.3)	10 (23.3)	0.628	16 (23.9)	5 (15.2)	0.465	6 (12.5)	8 (36.4)	0.095
Scale Tests (±SD)									
Perceived Stress Scale	13.7 (± 5.1)	19.2 (± 6.7)	0.000	14.8 (± 6.1)	18.6 (± 6.4)	0.006	14.2 (± 6.3)	19.2 (± 5.8)	0.002
PROMIS Fatigue	48.7 (± 7.7)	54.3 (± 6.6)	0.000	50.0 (± 7.8)	53.4 (± 6.9)	0.036	51.5 (± 8.4)	52.3 ± (4.9)	0.610
PROMIS Sleep Disturbance	46.0 (± 8.0)	54.3 (± 6.6)	0.386	46.7 (± 8.6)	46.3 (± 8.5)	0.831	47.8 (± 8.8)	47.4 (± 7.9)	0.851
Beck Depression	4.1 (± 3.4)	12.3 (± 7.6)	0.000	6.3 (± 5.8)	10.3 (± 8.3)	*0.017*	5.9 (± 5.3)	10.8 (± 7.5)	*0.002*
Patient Health Questionnaire	4.6 (± 3.2)	8.5 (± 4.4)	0.000	5.5 (± 3.8)	7.8 (± 4.6)	0.009	6.1 (± 3.9)	7.4 (± 3.7)	0.174
BPI Pain Rating	13.8 (± 9.2)	15.3 (± 11.0)	0.686	11.8 (± 8.6)	18.2 (± 11.3)	0.068	14.1 (± 9.5)	13.2 (± 11.6)	0.857
BPI Pain Interference	16.6 (± 13.8)	14.8 (± 11.8)	0.696	13.1 (± 11.8)	18.2 (± 12.8)	0.231	16.3 (± 13.4)	13.5 (± 15.0)	0.685
Fagerstrom	2.7 (± 2.0)	2.9 (± 2.6)	0.866	2.9 (± 1.9)	2.4 (± 3.4)	0.748	1.5 (± 1.6)	3.5 (± 1.9)	0.058
Age of First Cannabis Use (± SD)	17.1 (± 3.8)	17.6 (± 5.8)	*0.783*	17.5 (± 5.1)	17.0 (± 4.2)	*0.510*	17.8 (± 4.7)	18.1 (± 6.5)	*0.931*
Age of Onset of Regular Use (SD)	20.7 (± 6.3)	20.0 (± 6.3)	*0.296*	20.5 (± 6.7)	20.1 (± 5.3)	*0.807*	21.7 (± 7.2)	20.4 (± 6.7)	*0.192*
Reason for Medicinal Cannabis Use (%)									
Conventional treatments don’t work	9 (15.8)	11 (25.6)	0.226	11 (16.4)	9 (27.3)	0.202	7 (14.6)	9 (40.9)	0.015
My doctor recommended it to me	1 (1.8)	8 (18.6)	0.004	6 (9.0)	3 (9.1)	0.982	3 (6.2)	4 (18.2)	0.122
Internet search on treatments for my condition	5 (8.8)	10 (23.3)	0.045	8 (11.9)	7 (21.2)	0.222	6 (12.5)	5 (22.7)	0.275
Experiences Pain (%)	12 (21.1)	23 (53.5)	0.001	19 (28.4)	16 (48.5)	0.047	14 (29.2)	6 (27.3)	0.871
Condition (%)									
Anxiety/Stress	38 (66.7)	39 (90.7)	0.005	50 (74.6)	27 (81.8)	0.422	36 (75.0)	15 (68.2)	0.552
Depression	18 (31.6)	29 (67.4)	0.000	25 (37.3)	22 (66.7)	0.006	15 (31.2)	13 (59.1)	0.027
Insomnia/Sleep Issues	29 (50.9)	24 (55.8)	0.624	36 (53.7)	17 (51.5)	0.835	24 (50.0)	17 (77.3)	0.032
Bipolar	0 (0.0)	5 (11.6)	0.008	4 (6.0)	1 (3.0)	0.526	1 (2.1)	3 (13.6)	0.053
Dispensary Visits Per Month (± SD)	4.2 (± 3.5)	3.9 (± 3.4)	*0.664*	3.7 (± 3.3)	4.7 (± 3.7)	*0.151*	3.1 (± 3.1)	5.0 (± 3.6)	*0.002*
Cannabis Use Quantity, grams per week (± SD)	4.5 (± 4.8)	4.0 (± 4.6)	*0.497*	3.9 (± 3.2)	5.2 (± 7.0)	*0.911*	3.4 (± 3.3)	4.1 (± 2.0)	*0.038*
Cannabis Use Frequency, times per month (± SD)	50.2 (± 27.8)	42.9 (± 34.3)	*0.185*	47.7 (± 30.8)	45.6 (± 31.1)	*0.836*	39.5 (± 28.7)	59.3 (± 28.7)	*0.012*
Time of Use: On an as-needed basis (%)	14 (24.6)	23 (53.5)	0.003	22 (32.8)	15 (45.5)	0.219	18 (37.5)	8 (36.4)	0.927
Form of Consumption (%)									
Smoking	42 (73.7)	29 (67.4)	0.496	50 (74.6)	21 (63.6)	0.255	26 (54.2)	21 (95.5)	0.001
Vaporizer	22 (38.6)	20 (46.5)	0.427	26 (38.8)	16 (48.5)	0.356	23 (47.9)	5 (22.7)	0.046
Tincture	5 (8.8)	10 (23.3)	0.045	7 (10.4)	8 (24.2)	0.069	10 (20.8)	0 (0.0)	0.021
Capsules	6 (10.5)	11 (25.6)	0.047	6 (9.0)	11 (33.3)	0.002	8 (16.7)	1 (4.5)	0.160
Cannabis Product: CBD Capsules (%)	5 (8.8)	18 (41.9)	0.000	11 (16.4)	12 (36.4)	0.026	9 (18.8)	4 (18.2)	0.955
Concentration: Pure CBD (%)	6 (10.5)	12 (27.9)	0.025	9 (13.4)	9 (27.3)	0.09	10 (20.8)	2 (9.1)	0.226
Experienced Negative Effects with Cannabis Use (%)	24 (42.1)	30 (69.8)	0.008	34 (50.7)	20 (60.6)	0.392	18 (37.5)	13 (59.1)	0.106
Negative Effects (%)									
Short-term memory loss	10 (17.5)	15 (34.9)	0.047	17 (25.4)	8 (24.2)	0.902	7 (14.6)	8 (36.4)	0.039
Increased anxiety	16 (28.1)	20 (46.5)	0.057	24 (35.8)	12 (36.4)	0.958	12 (25.0)	11 (50.0)	0.039
Paranoia	8 (14.0)	18 (41.9)	0.002	16 (23.9)	10 (30.3)	0.491	6 (12.5)	9 (40.9)	0.007

## Discussion

In the present study, we conducted an in depth evaluation of the mental health of one hundred medical cannabis users at a community dispensary. By using a standardized, structured clinical interview combined with established self-report questionnaires, we were able to obtain detailed information about the prevalence of major psychiatric conditions, as well as symptom severity of depression and a range of other measures of mental health. These results were then combined with the extensive data on cannabis use to create a detailed profile of mental health in the dispensary population. Overall, we observed that lifetime prevalence of mental illness in this population was high, and a large proportion of subjects endorsed psychological symptoms. The proportion of subjects who met criteria for classification of a *current* major psychiatric disorder was low for mood disorders, but high for anxiety disorders and substance abuse/dependence.

Most importantly, the results of the MINI interviews, which includes criteria for 23 diagnostic psychiatric disorders from the DSM-IV [31], indicated that the lifetime rates of mental disorders were high compared to the general Canadian population. The 2012 Canadian Community Health Survey study determined that 33.1% of Canadians met the criteria for a major mental or substance use disorder at some point in their life [41]. This included 12.6% of Canadians meeting criteria for lifetime depression and 8.7% of Canadians meeting criteria for lifetime generalised anxiety disorder. In our study, 80% of the dispensary clients met criteria for a major lifetime psychiatric disorder – more than double that of the general population. Indeed, this is likely an underestimate, as the MINI only records current (but not past) anxiety and dependence disorders. Our cohort included 33% who had experienced major depression in their lifetime, and 16% who had current generalized anxiety disorder – again, rates that are much higher than in the general population. While the present study was not designed as an epidemiological study, there are no obvious demographic biases that are likely to account for the high rates of mental illness; for example, both lifetime depression and anxiety disorders were very common in subjects, yet the cohort was mostly male, who typically exhibit lower rates of mood and anxiety disorders than females [42–44]. The study was also conducted in one of the more affluent neighbourhoods of the city, and three quarters of subjects had some level of college education. Nevertheless, more epidemiologically robust studies in the future are required to draw firm conclusions about the prevalence of lifetime mental illness at dispensaries, compared to the general populace.

Current symptom severity was assessed for a spectrum of psychological symptoms using seven well validated, standardized self-report questionnaires. With the exception of the BDI, these questionnaires do not map directly on to a specific psychiatric disorder (although they may be important individual symptoms), and so should be seen as complementary to the findings from the MINI. They provide measures of mental health, including stress and sleep quality, which are not diagnostic conditions per se, but reflect common issues for psychological well-being, and are likely an important reason for use of medical cannabis. While the PROMIS scales for sleep and fatigue do not provide rankings, all of the other scales have specific cut-off scores which allow determination of whether an individual’s symptoms are in the “normal” category, or more severe. In this dispensary cohort, 62% endorsed “moderate” or greater perceived stress on the PSS10, 57% were above “minimal” ranking on the PHQ-15, 34% were above “normal” for the BPI, 17% were above “normal” on the BDI, and 11% had greater than “low” dependence on the FTND. Overall, 82% of participants scored above normal for at least one of these five questionnaires, and 55% scored above normal for two or more, indicating that psychological distress was common. These numbers also exclude the results from the two PROMIS questionnaires, and do not capture symptom severity related to anxiety or drug dependence (other than tobacco), which were two of the three most common conditions detected with the MINI. Thus, psychological symptoms of above normal severity were the norm in this population. Interestingly, however, few subjects achieved scores in the more severe categories of these five scales. This is consistent with the results of the MINI for mood disorders, where few subjects met the criteria for a current diagnosis. However, it is also worth considering that the cut-off values used in the present study, as provided by the test creators, have an unknown validity in this population of medical cannabis users, and future studies should determine the psychometric validity of these values.

Subjects’ self-reported reasons for using medical cannabis were in general agreement with the results of the MINI and questionnaires. When asked why they used medical cannabis, the top four reasons chosen from a check list included anxiety, sleep, depression and pain, consistent with reports from other check list dispensary studies [45–48]. Anxiety disorders were the most common MINI diagnosis in this cohort, and lifetime depression was also common. The standard MINI does not assess sleep or pain disorders, but these symptoms are commonly associated with substance dependence [49–51], which was a frequent MINI diagnosis (40%). Importantly, for both anxiety and depression, a substantially greater proportion of subjects selected this option from the self-report checklist than met criteria for a current or lifetime disorder. It is therefore likely than many subjects experience symptoms associated with these disorders, but of a milder severity or fewer in number than required for a DSM or ICD diagnosis. The results from the standardized questionnaires support this hypothesis, as many subjects endorsed symptoms of stress, pain and depression above normal, but not in the more severe categories. Subjects with milder symptoms might therefore experience barriers to accessing pharmaceutical treatments, or may find a better balance between therapeutic benefits and side-effects with medical cannabis. Additionally, a proportion of these subjects may actually be manging their symptoms effectively with cannabis. Indeed, only 23% of subjects self-reported pharmaceutical treatments, including past or current use of antidepressant, anxiolytic, or antipsychotic medication, despite the high rate of psychiatric diagnoses within our population.

Overall cannabis use in the dispensary cohort reflected a broad range of behaviour. Subjects differed widely in the amount of cannabis consumed and how frequently it was ingested. While most people smoked cannabis, and preferred to use the dried plant product, a significant proportion of individuals consumed other forms, such as capsule and tinctures. Our exploratory analyses indicated that patterns of medical cannabis use differed, depending on the specific psychiatric disorder involved. This included the route of administration, when the product was used in relation to symptoms, and the CBD:THC ratio. It is therefore important for cannabis studies to not treat medical cannabis users as a homogeneous group [52, 53], as individuals appear to tailor their use depending on the psychiatric condition they are trying to treat. While the present study was not able to determine the clinical efficacy of cannabis products, it does provide important insight into the symptoms and patterns of use that everyday medical cannabis consumers use to treat their symptoms. Many of these symptoms are in the mild-to-moderate range, and it is therefore likely than many individuals are able to find some relief through self-medication with cannabis [54]. Additional important information, presently not collected, would have been to determine who prescribed the medical cannabis to clients (physician, nurse practitioner or naturopath) to determine if this differed in terms of cannabis use or mental health condition.

The present study has a number of limitations. Firstly, as noted above, the sample was not selected using standard epidemiological techniques, and so extrapolation to the general population as a whole is not valid. Nevertheless, the subjects were chosen at random, and so it is probably representative at least of the types of individuals who use cannabis dispensaries. Secondly, the modest size of the cohort means that it was not possible to compare extensively details about cannabis use between all of the different psychiatric diagnoses that can be determined with the MINI. For anxiety disorders, depression and drug dependence, there were sufficient numbers to validly compare symptom severity and patterns of cannabis use, but for others (such as post-traumatic stress disorder) a larger number of subjects would have been needed. Thirdly, several participants reported medical cannabis use to treat focus and attention disorders, such as attention deficit hyperactivity disorder (ADHD), but a diagnostic test is not included in the MINI neuropsychiatric test version 6. Fourthly, anxiety symptom severity was not measured using an objective and validated questionnaire, such as the Generalized Anxiety Disorder 7-item (GAD-7) form.

## Conclusions

In summary, the findings of the current study indicate that both lifetime and current psychiatric illness occur with a high prevalence in medical cannabis users. Distressful psychological symptoms were also endorsed by a large proportion of the population, although these may often be of a lower intensity than needed for a psychiatric diagnosis. Results were determined with the use of a structured clinical assessment combined with standardized symptom severity questionnaires, similar to those used in clinical drug trials. This rigorous data adds to the extant literature on mental illness in dispensaries, which is largely based on check list surveys (although see [45]). Future studies using the same rigor of psychiatric evaluation but with larger sample sizes will allow detailed patterns of medical cannabis use to be determined for a wider range of disorders, and better determine the potential benefits of medical cannabis for mental illness.

## Data Availability

The datasets generated and/or analysed during the current study are not publicly available due to the sensitive nature of clinical information about mental health and drug use, but may be available in collaboration with the corresponding author on reasonable request.

## Abbreviations

ADHD: Attention Deficit Hyperactivity Disorder
BDI: Beck Depression Inventory
BPI: Brief Pain Inventory
CBD: Cannabidiol
DSM-IV: Diagnostic and Statistical Manual of Mental Disorders, 4th edition
FTND: Fagerström Test for Nicotine Dependence
GAD-7: Generalized Anxiety Disorder 7-item
ICD-10: International Classification of Diseases, 10th edition
MDD: Major Depressive Disorder
MDE: Major Depressive Episode
MINI: Mini-International Neuropsychiatric Interview
PHQ-15: Patient Health Questionnaire 15-item
PROMIS: Patient Reported Outcomes Measurement Information System
PSS10: Perceived Stress Scale 10-item
SPSS: Statistical Package for the Social Sciences
THC: Tetrahydrocannabinol
